# A novel technique for detoxification of phenol from wastewater: Nanoparticle Assisted Nano Filtration (NANF)

**DOI:** 10.1186/s40201-016-0249-8

**Published:** 2016-05-24

**Authors:** L. D. Naidu, S. Saravanan, Mukesh Goel, S. Periasamy, Pieter Stroeve

**Affiliations:** Department of Chemical Engineering, National Institute of Technology, Tiruchirapalli, 15 India; Center for Environmental Engineering, PRIST University, Thanjavur, India; Department of Textile Technology, PSG College of Technology, Coimbatore, India; Department of Chemical Engineering, University of California Davis, Davis, CA 95616 USA

**Keywords:** Phenol, Nanoparticle, Nanofiltration, NANF, COD, Nanoporous membranes

## Abstract

**Background:**

Phenol is one of the most versatile and important organic compound. It is also a growing concern as water pollutants due to its high persistence and toxicity. Removal of Phenol from wastewaters was investigated using a novel nanoparticle adsorption and nanofiltration technique named as Nanoparticle Assisted Nano Filtration (NANF).

**Methods:**

The nanoparticle used for NANF study were silver nanoparticles and synthesized to three distinct average particle sizes of 10 nm, 40 nm and 70 nm. The effect of nanoparticle size, their concentrations and their tri and diparticle combinations upon phenol removal were studied.

**Results:**

Total surface areas (TSA) for various particle size and concentrations have been calculated and the highest was 4710 × 10^12 ^nm^2 ^for 10 nm particles and 180 ppm concentration while the lowest was for 2461 × 10^11^ for 70 nm and 60 ppm concentrations. Tri and diparticle studies showed more phenol removal % than that of their individual particles, particularly for using small particles on large membrane pore size and large particles at low concentrations. These results have also been confirmed with COD and toxicity removal studies.

**Conclusions:**

The combination of nanoparticles adsorption and nanofiltration results in high phenol removal and mineralization, leading to the conclusion that NANF has very high potential for treating toxic chemical wastewaters.

## Background

Water is the central element of all vital social and economic processes. Because of the development of consumer society, harmful chemicals are being generated in huge quantities throughout the world. The problems derived from the toxicological effects of these organic compounds must be resolved for the benefits of entire society. The problem is certainly complex and it is imperative that novel procedures are required to deal with this extensive range of tribulations. There are ample treatment technologies for sewage, distillery effluents and so on, which contain biodegradable organics, but not so much for toxic effluents containing xenobiotic compounds, which are often non-biodegradable or only partially biodegradable. Biological treatment though promises much in this regard is handicapped by its slow oxidation characteristics and incomplete mineralization of toxic chemicals [1–5].

Nanotechnology has also attracted the versatile membrane filtration process for water treatment. Nanofiltration and Reverse Osmosis (RO), are two common membrane filtration processes for toxic chemical removal from wastewaters and have been successfully applied in removing BOD/COD from many wastewaters [6–9]. The powerful membrane technology is however, limited by high costs associated with it and other operational considerations. One of the significant ways to overcome this limitation is to couple membrane separation with another technology. An interesting solution is proposed by some researchers; polymer enhanced ultrafiltration (PEUF) [10, 11]. This was especially found to be more relevant in removing heavy metals from aqueous solutions. It combines UF with metal complexation using water-soluble polymers. The formed complexes are sufficiently large to be retained by a UF membrane. There has been several reports on the use of PEUF for treatment of wastewaters. This is however, limited to metal removal and has not found applications in removing toxic chemicals from water and wastewater [12, 13].

A modified version of this technology is called Nanoparticle Assisted Nanofiltration (NANF); nanofiltration in conjunction with adsorption using nanoparticles. The large surface area of nanoparticles makes them a potent adsorbent for toxic chemical removal. The first step in the process is adsorption of toxic organic chemicals on nanoparticles, followed by filtration with a NF membrane, which permits the passage of water and other smaller compounds, but retaining the toxic organic compounds. For polluted water, the full treatment steps conventionally adopted in practice includes pre-oxidation, enhanced coagulation, sedimentation, sand filtration, main oxidation, GAC filtration, etc. Such a treatment chain is however too long to be afforded for developing countries. Therefore NANF, using nano-adsorbents, is a suitable alternative toconventional wastewater treatment plants in dealing with toxic chemicals. In this work, silver nano-particles have been tested for their ability to adsorb organic compounds, which are then retained with a NF membranes. Phenol is used as the model organic compound for these experiments because it is one of the major waste-products in a wide range of manufacturing industries, e.g., chemical and pharmaceutical, paintsand textiles, paper and pulp, plastics and polymer, oil and gasoline as well as coking ovens and metallurgical furnaces [14, 15]. Three different nanoparticles sizes, 10, 40 and 70 nm, and four different NF membranes of diameter, 10, 30, 50 and 80 nm were used for the experiments.

## Methods

### Adsorbent

Silver nanoparticles of different sizes were synthesized using a chemical reduction method [16]. Dynamic light scattering (DLS, Brookhaven Instruments Corporation, USA) was used to analyze the size of the nanoparticles.

### Adsorbate

Phenol was obtained from Merck, and its 500 ppm stock solution was prepared in double distilled water. Solution of 200 ppm phenol used in the experiments was prepared from stock solution and double distilled water was used for necessary dilutions. All reagents used in the investigation were of analytical grade.

### Analytical measurements

Phenol was analyzed using the Amino-Antipyrine method [17]. For phenol analysis, 3 ml of distilled water was added with 1 ml of centrifuged sample from each reactor to make the sample to 4 ml. The sample vial was added with 0.60 ml of K_3_ Fe CN_6,_ 0.20 ml of NH_4_OH and 0.60 ml of 4-amino antipyrine. The absorbance of the sample was measured with UV–VIS spectrophotometry (Lab India). Theoretical density values were measured at 406 nm to yield the concentration of phenol. Chemical oxygen demand (COD) was measured using a HACH Colorimeter (DR 890). The COD solution (HR grade – 0–1500 ppm) was prepared by mixing 0.25 ml of COD solution A and 2.8 ml of COD solution B. To this solution, 2 ml of centrifuged sample (include dilution) was added. The digestion was done at 1500 C in a HACH COD digester for two hours using HACH COD vials. Final COD value after air cooling was measured in a HACH-DR/890 colorimeter. The toxicity of phenols before and after treatments was realized using Resazurin reduction method [18]. Pre-cultivated *Bacillus cereus* culture *w*as used as the test organisms and was cultivated on nutrient broth medium. Resazurin changes color in the presence of dehydrogenase enzyme activity resulting from microorganisms actively growing in a culture medium. In the presence of an active bacterial culture, resazurin changes color from blue to pink. If bacterial growth is inhibited, no reduction of the resazurin occurs, and such a sample would remain blue. The reaction time for assay was 20 min and would be inhibited by the addition of 50 μl of HgCl_2_. The colour intensity of centrifuged samples is determined using the UV–VIS spectrophotometer at 610 nm. Resazurin solution was prepared by dissolving 1 tablet of resazurin (5 mg in 100 ml) in 100 ml of distilled water. The resazurin solution was stored in a brown bottle and kept in a temperature of 4 °C.

### NANF experiments

**Fig. 1:**
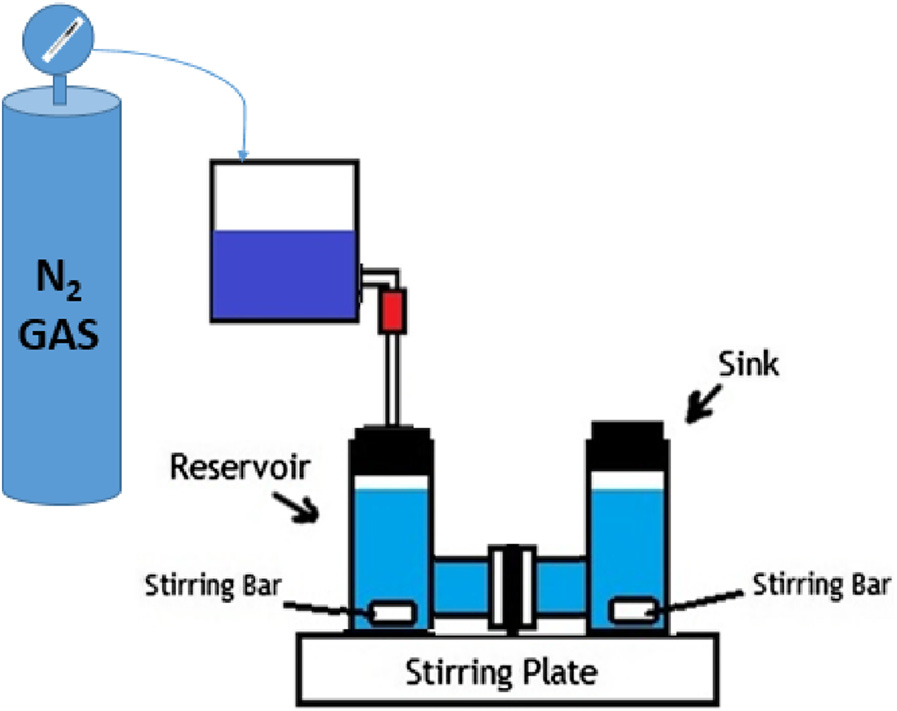
Schematic diagram of Experimental Set up

All experiments were conducted at 200 ppm phenol concentration and at pH 7 by the addition of NaOH or HCl. Experiments were conducted in a stirred membrane reactor. The schematic of the reactor is shown in Fig. 1. The details of the reactor is given elsewhere [19]. The effective permeation area of the NF membrane was 1.77 cm^2^, and the volumes of both compartments were 35.0 mL. Mixing was obtained using magnetic stirrers, which were present in both compartments.

### Experimental procedure

Commercial PCTE membranes (Poretics, Inc.) were used as NF membranes [20]. Membranes are numbered according to their diameter. Membrane with 10 nm pore size was referred as M1, whereas, membranes with 80 nm pore size was referred as M4. The intermediate membranes were referred as M2 and M3 respectively. The pressure is obtained through compressed N_2_ gas. One hour before experiment, 0.5 mL of nanoparticles solution was mixed with 35 mL of neutral solution containing 200-ppm phenol. The solution was then moved to the membrane reactor. Additionally, each experiment was conducted without any nanoparticles to comprehensively evaluate the potential adsorption and filtration performance of NANF.

### Theoretical surface area calculation

The surface area calculation was performed as given below.

- Surface area of a single nanoparticles of 10 nm diameter (5 nm radius) is given by
  $$ \begin{array}{l}=4\times 3.14\times {5}^2\ \mathrm{n}{\mathrm{m}}^2\\ {}=314\ \mathrm{n}{\mathrm{m}}^2\end{array} $$

b) Number of particles is obtained from
  i) Concentration of the nanoparticles solution, i.e. 60, 120 and 180 ppm
  ii) Nanoparticle average size
  iii) Total volume of nanoparticle solution used, i.e. 0.5 ml
  iv) Density of silver metal (10.49 g/cc)

From i), we calculate the total weight of the nanoparticles used for adsorption, for example60 ppm means 60 g in 10^6^ ml of water, so for 0.5 ml of water

$$ \begin{array}{l}=60\times 0.5\kern0.05em /\kern0.05em {10}^6\\ {}=30\times {10}^{-6}\ \mathrm{g}-------\mathrm{x}\kern1em \end{array} $$

From ii) we can calculate the volume of individual particles, for example for 10 nm particle

$$ \begin{array}{l} = 1.33 \times 3.14 \times {5}^3 \times {\left(1{0}^{\hbox{-} 7}\right)}^2\mathrm{c}{\mathrm{m}}^3\\ {} = 523 \times 1{0}^{\hbox{-} 21}\mathrm{c}{\mathrm{m}}^3\end{array} $$

Weight of an individual particle can be calculated from the density value of silver metal (10.49 g/cc)

$$ \begin{array}{l} = 10.49 \times 523 \times 1{0}^{\hbox{-} 21}/\ 1\\ {} = 548 \times 1{0}^{\hbox{-} 20}\mathrm{g}\ \hbox{-} \hbox{-} \hbox{-} \hbox{-} \hbox{-} \hbox{-} \hbox{-}\ \mathrm{y}\kern0.5em \end{array} $$

Hence, total number of particles can be obtained from

$$ \begin{array}{l} = \mathrm{x}/\mathrm{y}\hfill \\ {} = 30 \times 1{0}^{\hbox{-} 6}\mathrm{g}\ /\ 548 \times 1{0}^{\hbox{-} 20}\mathrm{g}\hfill \\ {} = 3000 \times 1{0}^{12}/\ 548\hfill \\ {}\begin{array}{l} = 5.47 \times 1{0}^{12}\\ {} \approx 5 \times 1{0}^{12}\end{array}\hfill \end{array} $$

c) Total surface area is obtained by
  $$ \begin{array}{l} = \mathrm{Surface}\ \mathrm{area}\ \mathrm{of}\ \mathrm{individual}\ \mathrm{particle} \times \mathrm{Total}\ \mathrm{n}\mathrm{umber}\ \mathrm{of}\ \mathrm{particle}\mathrm{s}\\ {} = 314\ \mathrm{n}{\mathrm{m}}^2 \times 5 \times 1{0}^{12}\\ {} = 1570 \times 1{0}^{12}\mathrm{n}{\mathrm{m}}^2\end{array} $$

## Results and discussion

The current study combines two materials, i.e. the membrane filters and the nanomaterials, to exploit their inherent characteristics for the removal of toxic materials from water. For this purpose, membranes of various pore sizes are used along with silver nanoparticles with various average particle sizes.

### Silver nanoparticles

**Fig. 2:**
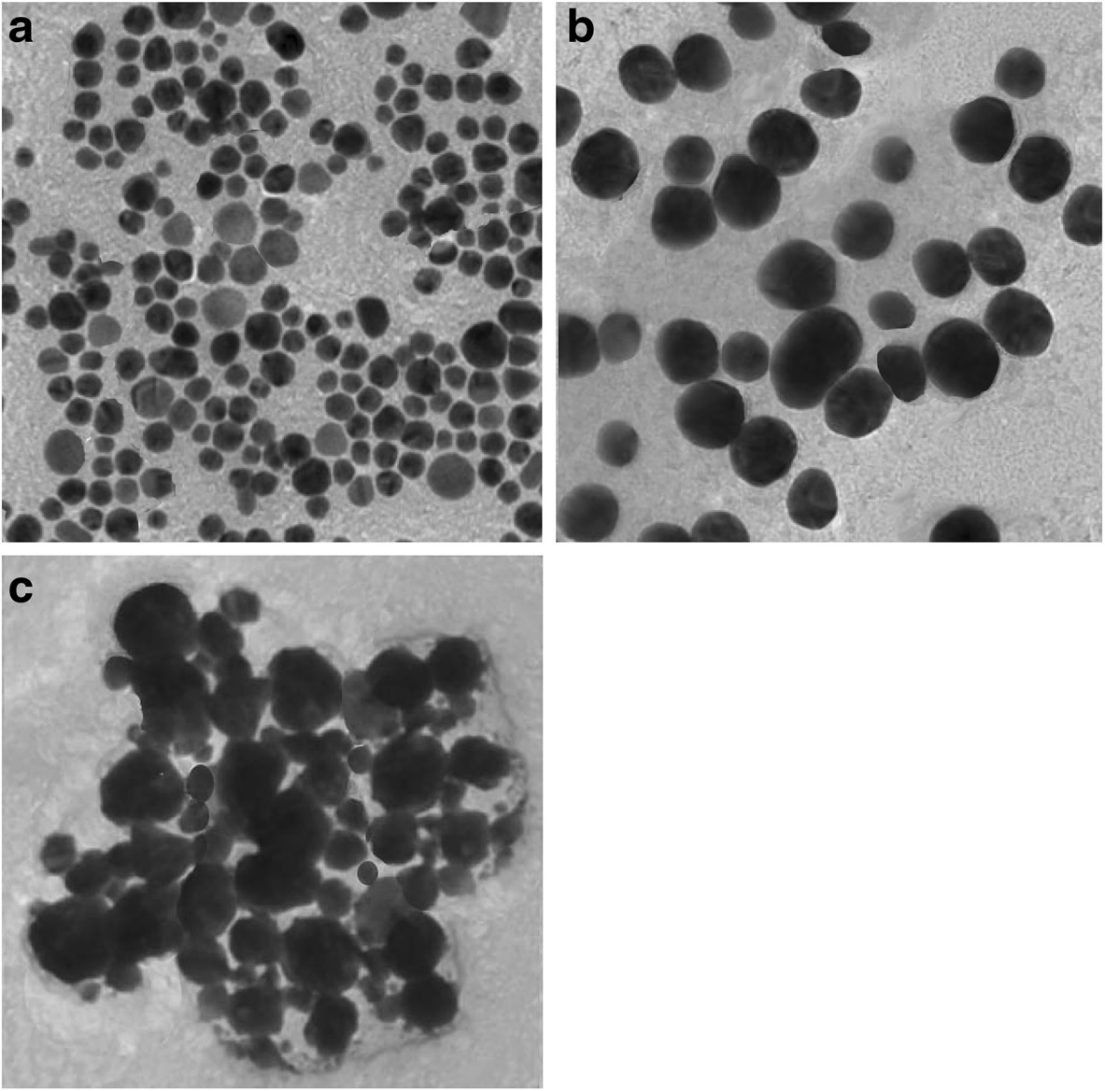
Transmission electron micrograph of silver nanoparticles with average particle size of **a**) 10 nm, **b**) 40 nm and **c**) 70 nm

Figure 2 shows the transmission electron micrograph of the silver nanoparticles, which were prepared using three distinct combinations of the bottom-up nanoparticles synthesis process for obtaining nanoparticles of three different average nanoparticles sizes i.e. 10 nm (Fig. 2a), 40 nm (Fig. 2b) and 70 nm (Fig. 2c). Silver nanoparticles with average size of 10 nm were obtained with 10 °C synthesis temperature, Polyvinyl Pyrolidone (PVP) as the dispersing and stabilizing agent and hydrazine as the reducing agent; 40 nm were obtained with 40 °C synthesis temperature, PVP and hydrazine; and 70 nm were obtained with 40 °C synthesis temperature, oligo condensate of naphthalene sulfonic acid (OCNS) and D-glucose combinations. It can be observed from the transmission electron micrographs (Fig. 2) that the particles have been formed with proper geometrical shape, almost spherical for most particles. It can be observed that the particles prepared with PVP have less variation in the particle size distribution (narrow) while those prepared with OCNS comparatively have a  wide particle size distribution (broad). The mechanism of such particles formation with varying average particle size and distribution are discussed elsewhere [13].

### Nanoparticle assisted nano filtration (NANF) effect on phenol removal from water

**Fig. 3:**
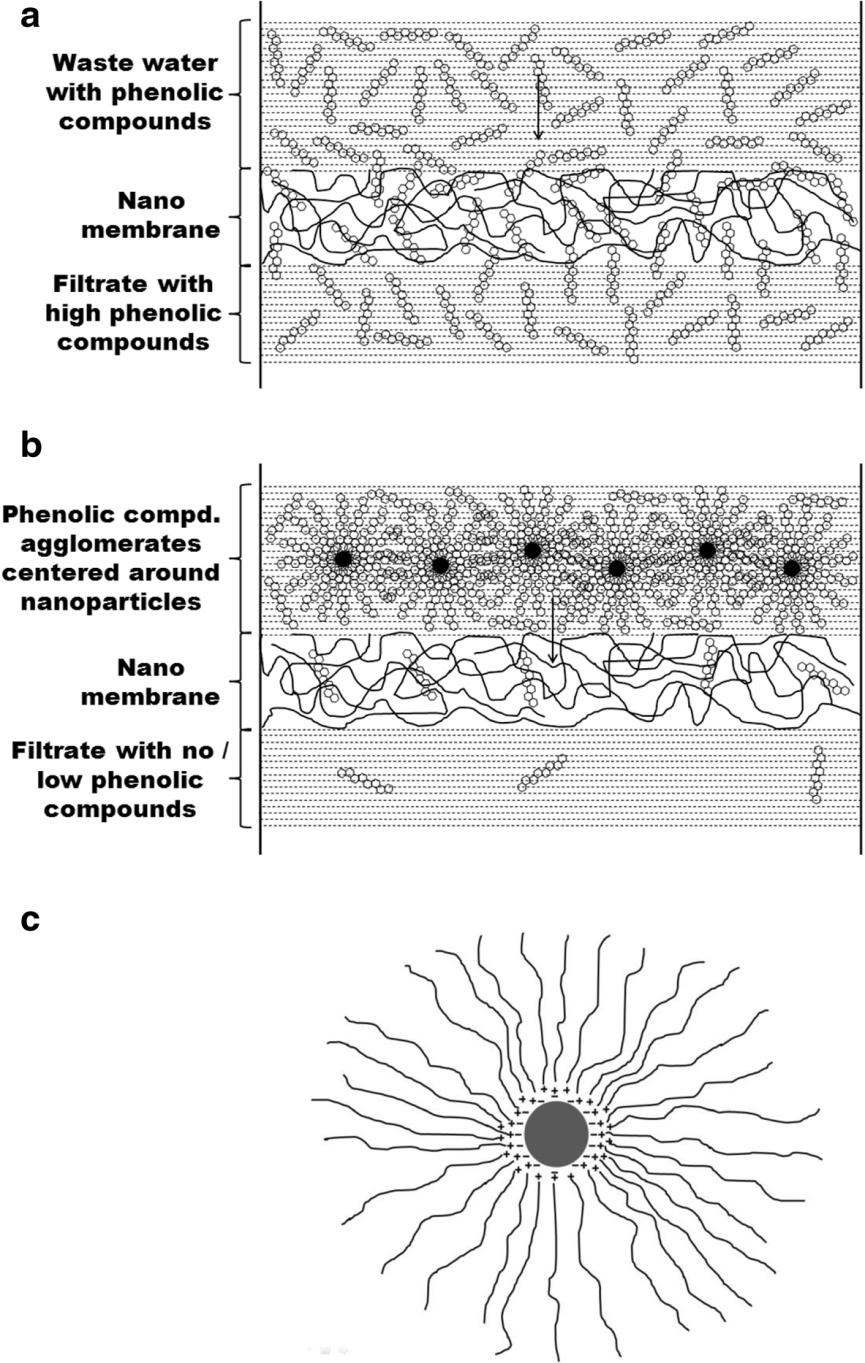
Schematic of filtration of model phenolic compound through nano membrane **a**) without nano particles, **b**) with nanoparticles (NANF) and **c**) electrostatic adsorption

The work started with adsorption of phenols using silver nanoparticles. Since three distinct average size particle samples were prepared, the three particles batches are used in equal proportion, with the total particle concentrations at 60 ppm. Gulrajani et al. [16] have shown that at 60 ppm particle concentration good particle performance was obtained in their study. The effects of nanoparticle size and membrane pore size on filtration of the phenol are presented in Table 1. It can be observed from Table 1 that for all particle sizes the presence of the nanoparticles show a significant improvement in filtering the phenol with reference to the respective counterpart, i.e. in the absence of the particles for the respective membrane. The results clearly show the effectiveness of the NANF process for phenol removal. As high as 675 % improvement is observed for NANF process (membrane filtration with nanoparticles), compared to the process without nanoparticles. These results can be explained based on the mechanism of phenol adsorption followed by agglomeration of the nanoparticles, which results in a net increase in the particle size, which then cannot pass through the membrane pores and hence are filtered. Such effect is schematically shown in Fig. 3. In the absence of nanoparticles (Fig. 3a) the molecular phenolic compounds can easily pass through the pores of the membranes by diffusion. However, in the presence of nanoparticles (Fig. 3b) whose surface area is very large, there exists a spontaneous attraction of phenolic compounds towards the surface of the particles due to i) electrostatic charge attraction between the nanoparticles and the phenolic compounds (Fig. 3c) and ii) aggregate formation to obtain a lower energy state of the solute and the squeezing out of solvent in support of solute separation [21].

**Table 1:** Phenol removal percentage with and without nanoparticles for mixtures of nanoparticles

Membrane size (nm)	Phenol Removal (%)		% improvement
	Nanoparticles	No Nanoparticle	
10	68	27	152
30	57	18	216
50	42	11	281
80	31	4	675

The effect of membrane pore size on NANF is observed to be significant. Filtration effects are large for the 80 nm pore size membranes (M4), i.e. up to a 675 % improvement (obtained from the phenol removal % with the presence and absence of nanoparticles [((31–4) × 100)/4]). It is surprising to note that such % improvement is low for 10 nm pore size membranes (M1), i.e. only a 151 % improvement. This may be explained to the fact that for large pore size almost all the phenolic compounds pass through the pores in the absence of the particles and the phenol removal % is very low; but with the low value in the denominator, for the case of the presence of nanoaparticles, the % improvement becomes large, as shown in the calculation above. However, in contrast with the small pore size membrane, i.e. 10 nm, there is a considerable amount of phenol removal even in the absence of particles, i.e. 27 %. Hence, even though the actual amount of phenol removal is high in the combined nanoparticles and membrane approach, i.e. 68 %, the % improvement in the presence of nanoparticles is only 151 %. It can be noticed that the best results are obtained for the small pore size membrane (10 nm) and the nanoparticles combinations. This can be explained to the fact that the nanoparticles have enormous surface area particularly below the 100 nm size. The specific surface area of the particles increases exponentially with decreasing size (Theodore, 2005). Hence, availability of large surface areas contributes to surface adsorption of the toxic compounds, which are then filtered by membrane filtration. The low phenol removal %, i.e. 31 % with the 80 nm membrane, even in the presence of the nanoparticles, is due to the presence of nanoparticles whose size is less than 80 nm. These particles pass through the pores resulting in lower nanoparticles concentration and a consequent decrease in phenol removal. This indicates that the smaller particles play an important role in adsorption and hence the phenol removal %. The effectiveness of the hybrid process is also reported by Geckeier et al. [22]. They studied polymer enhanced ultrafiltration (PEUF), which was helpful in removing metal from wastewater by complexing of polymers with the metals and subsequent removal from the wastewater. Similar study was reported by Li et al. [23].

### Effect of concentration and particle size of silver nanoparticles

**Fig. 4:**
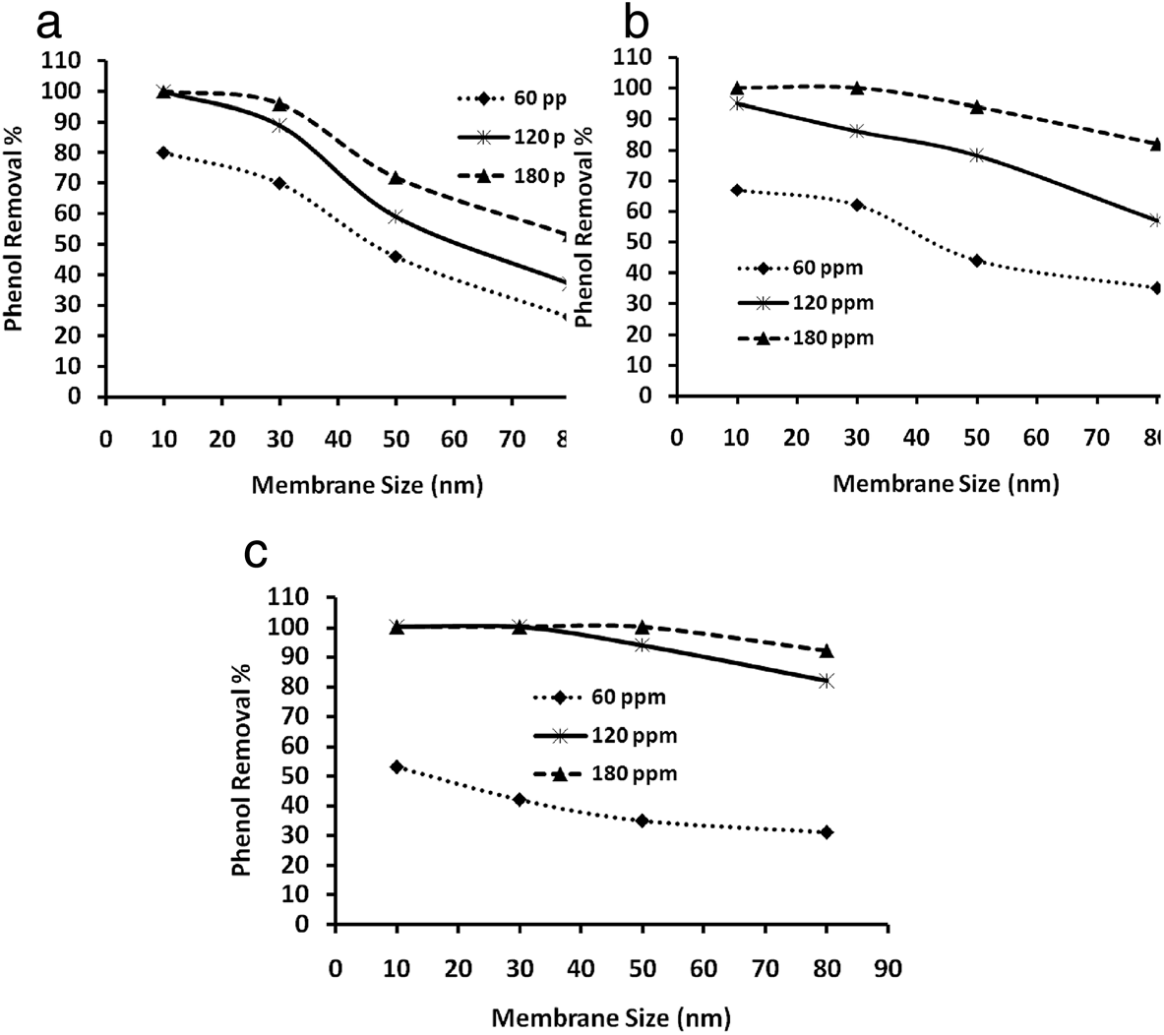
Phenol removal %, through membranes having various average pore sizes, as an effect of concentration of silver nanoparticles of **a**) 10 nm, **b**) 40 nm and **c**) 70 nm

**Fig. 5:**
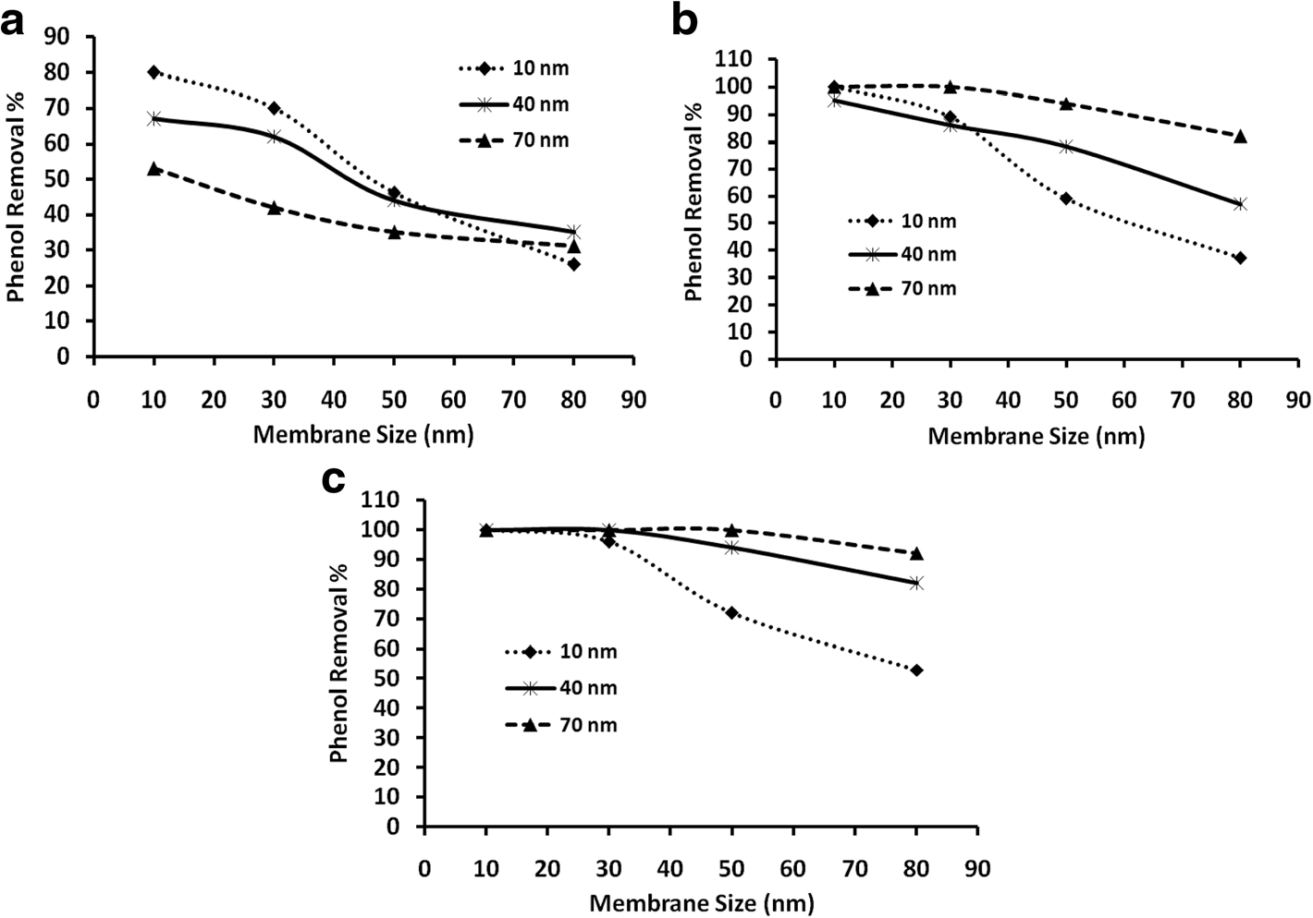
Phenol removal %, through membranes having various average pore sizes, with silver nanoparticles as an effect of the particles sizes, **a**) 60 ppm, **b**) 120 ppm and **c**) 180 ppm

Silver nanoparticles with three different average particle sizes, i.e. 10, 40 and 70 nm, each with three different particle concentrations, 60, 120 and 180 ppm, are used for phenol removal study on membranes (M1 – M5). The results are depicted in Fig. 4 and in Fig. 5. Figure 4a shows the effects of the three particle concentrations, for 10 nm silver nanoparticles. Similarly, Figs. 4b and c show the effects of the three particle concentrations for 40 and 70 nm nanoparticles, respectively. Inversely, Fig. 5 shows the effect of particle size for a given concentration, i.e. Figure 5a shows the effect of the three nanoparticles sizes for the 60 ppm particle concentration. Similarly, Figs. 5b and c  show the effect of the three nanoparticles size for the 120 and 180 ppm concentrations, respectively.

From Figs. 4-5, it can be inferred that for the 10 nm pore size membrane that about 100 % phenol removal is obtained for 120 and 180 ppm concentrations for all the three nanoparticles sizes. Further, it can be observed that for the 60 ppm particle concentration that, for almost for all particle sizes (Fig. 5a), the results are less than 100 % phenol removal on the 10 nm pore size membrane. Further, it can be observed that the phenol removal % decreases with increase in pore size of the membrane for almost all particle sizes, but with a varying slopes (Fig. 4a to c). The decrease is sharp after the 30 nm membrane and is well noticed for 60 ppm particle concentration (Fig. 4a to c and Fig. 5a). However, for higher particle concentrations, the decrease in phenol removal % with increasing membrane pore size is low. For the 10 nm size nanoparticles, a notable decrease is observed both for 120 (Fig. 5b) and 180 ppm (Fig. 5c) above the 30 nm membrane size. However, the decrease is less for the particle size of 40 nm both for 120 (Fig. 5b) and 180 ppm (Fig. 5c) after the 30 nm membrane size and is even less, almost reaching 100 % for the particle size of 70 nm both for 120 (Fig. 5b) and 180 ppm (Fig. 5c) above the 30 nm membrane size (Fig. 4c).

**Fig. 6:**
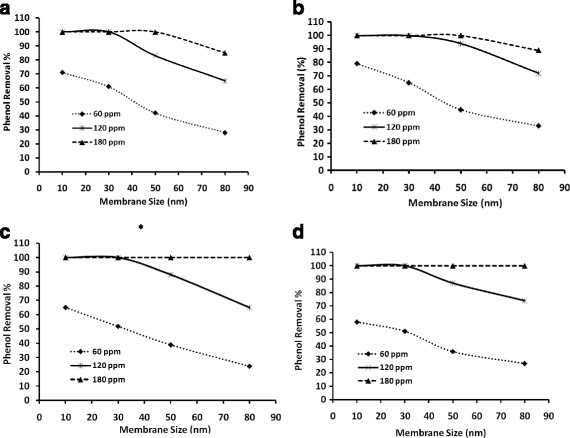
Phenol removal % through membranes having various average pore sizes with mixture of nanoparticles, **a**) 1:1:1 (10 nm, 40 nm, 70 nm), **b**) 1:1 (10 nm, 40 nm), **c**) 1:1 (10 nm, 70 nm) and **d**) 1:1 (40 nm, 70 nm)

These observations can be explained based on nanoparticles size, membrane pore size and the surface area available of the nanoparticles for adsorption [24]. The surface area of the individual nanoparticles, total number of nanoparticles available for a given particle size and concentration and hence the total surface area (TSA) available for the adsorption studies have been discussed previously and the values are presented in Table 2. It can be observed from Table 2 that the highest TSA is obtained for the 10 nm particle size at 180 ppm concentration, i.e. 4710 × 10^12^ nm^2^, and the lowest TSA of 2461 × 10^11^ nm^2^ for the particle size of 70 nm at 60 ppm concentration with the total % difference of 1813 [(4710 × 10^12 -^ 2461 × 10^11^) × 100/2461 × 10^11^]. For the other particle sizes and concentrations, the TSAs of the particles lie in between these two limits. Additionally, it can be observed from Table 2 that for the 60 ppm concentration the TSA for 10, 40 and 70 nm particles are 1570 × 10^12^, 4521 × 10^11^ and 2461 × 10^11^, respectively, and so on for the other concentrations. The percentage increases in TSA between the particles with various concentrations are shown separately in Table 3. The general formula for calculating % increase in TSA between various samples is shown as a foot note under Table 3. It can be observed from Table 3 that between samples 1& 4 (between10 and 40 nm both at 60 ppm conc.) the TSA increase is 247 % while between samples 4 & 7 (between 40 and 70 nm both at 60 ppm conc.) the TSA increase is only 84 %. However, the TSA increase between samples 1 & 7 (between 10 and 70 nm both at 60 ppm conc.) is a maximum of 537 %. Further it could be carefully noted that similar comparisons for the higher concentrations, i.e. 120 ppm and 180 ppm, also result in similar TSA % increases i.e. 247 %, 84 % and 537 %. However, in such trials when compared for the smallest particle (10 nm) with highest concentration (180 ppm) (sample 3) and largest particle (70 nm) at the lowest concentration (60 ppm) (sample 7) the TSA increase is the highest of all, about 1813 %.

**Table 2:** Theoretical calculation of total available surface area of the silver nanoparticles

Sample	Particle size (nm)	Surface Area (nm^2^)	Con. in ppm	No. of Particles	Total Surface Area (TSA) (nm^2^)	Comments
1	10	314	60	5 × 10^12^	1570 × 10^12^	Equal to 6
2	10	314	120	10 × 10^12^	3140 × 10^12^	In between
3	10	314	180	15 × 10^12^	4710 × 10^12^	Highest
4	40	5024	60	9 × 10^10^	4521 × 10^11^	Equal to 8
5	40	5024	120	18 × 10^10^	9043 × 10^11^	In between
6	40	5024	180	27 × 10^10^	1356 × 10^12^	Close to 1
7	70	15386	60	16 × 10^9^	2461 × 10^11^	Lowest
8	70	15386	120	32 × 10^9^	4923 × 10^11^	Close to 4
9	70	15386	180	48 × 10^9^	7385 × 10^11^	Close to 5

**Table 3:** Percentage increase in total surface area (TSA) between particles of various sizes and concentrations

Between Sample # and #	Increase in TSA (%)^a^
1 & 4	247
4 & 7	84
1&7	537
2&5	247
5&8	84
2&8	537
3&6	247
6&9	84
3&9	537
3 & 7	1813

^a^(TSA of 1 – TSA of 2) × 100/TSA of 2

It can be observed from Figs. 4-6 that the 10 nm particles at 180 ppm concentration with the 10 nm pore membrane result in 100 % phenol removal (Fig. 4a), while the 70 nm at 60 ppm with the 10 nm pore size membrane results in phenol removal of only about 50 % (Fig. 4c), which can be attributed to the large difference in the total surface areas available for adsorption, between these two samples, i.e. 1813 % difference. However, the phenol removal % for 10 nm particles on the larger pore size membranes, shows less than 100 % and is dramatic above the 30 nm pore size for all three concentrations (Fig. 4a), which could be because of the loss of nanoparticles through the large pore size membranes. Contrastingly, with 70 nm particle such decrease is observed to be low for 60 ppm and very low for the higher concentrations i.e. 120 and 180 ppm (Fig. 4c). In fact, at these concentrations almost 100 % phenol removal is observed for 70 nm particles except with the 80 nm pore size membrane, which may be due to some loss of the 70 nm particles with the 80 nm pore size membrane. The observations of phenol removal by the other particle sizes and concentrations lie in between these two limits of smallest particle and highest concentrations with the smallest membrane pore size (Fig. 4a & Fig. 5c) and the largest particle and lowest concentration with the largest membrane pore size (Fig. 4c & Fig. 5a). The results can be attributed to the corresponding TSA along with the loss of particles through the large pore size membrane, i.e., small particles of high concentrations might have large TSA but fail to show 100 % phenol removal with the large pore size membranes, which might be by virtue of their small size because they pass through the membrane pores and are lost (Fig. 4a & Fig. 5a to Fig. 5c).

The effect of the particle size upon phenol removal can be realized at 60 ppm concentration and with the 10 nm pore size membrane (Fig. 5a) where the loss of small particles should be very low. It can be observed from Fig. 4a that the 10, 40 and 70 nm size particles give about 82 %, 68 % and 52 % phenol removal which might be due to the difference in the TSA i.e. 1570 × 10^12^ nm^2 ^(sample 1), 4521 × 10^11^ nm^2 ^(sample 4) and 2461 × 10^11^ nm^2 ^(sample 7) for 10, 40 and 70 nm size particles, respectively. The corresponding % TSA increase between the samples are found to be 247 %, 84 % and 537 % for sample 1 & 4, sample 4 & 7 and sample 1 & 7, respectively. Though % increase between sample 1 & 7 is very large, the % phenol removal is only about 82 % for the 10 nm particles as against 52 % for the 70 nm particles. This is because the 10 nm particle is only the average size and so there may be particle loss for those whose size is smaller than 10 nm. This is also reported by Sarkar and Acarya [25]. They observed that phenol removal was maximum at lower particle size. Furthermore, Roostaei and Tezel [26], noted that adsorption capacity decreased by increasing the particle size. It can however, be concluded that the particle loss through the membrane impacts the phenol removal even though small size particle yields high TSA.

### Effect of nanoparticles mixtures

In the individual particle study discussed  in the previous section, either there was a particle loss effect for small particles or there was a low total surface area (TSA) effect for large particles which were particularly noticed at 60 ppm concentration. Hence we studied the effect of particle mixtures in various proportions for three concentrations, 60 ppm, 120 ppm and 180 ppm, for phenol removal. Results of the studies are shown in Fig. 6. Figure 6a depicts the effect of the three different particles i.e. 10 nm, 40 nm and 70 nm mixtures in equal proportions (1:1:1) for the three different concentrations. Accordingly 20 ppm of 10 nm particles, 20 ppm of 40 nm particles and 20 ppm of 70 nm particles were taken for forming a silver nanoparticles mixture solution. Similarly this was done for the other two concentrations i.e. for 120 ppm 40 ppm of each particle solutions and for 180 ppm 60 ppm of each particle solutions were taken. The effect of equal proportions (1:1:1) of the three nanoparticles (triparticle mixture) for three concentrations on various membrane are shown in Fig. 6a. Similarly, the other possible proportions of particles i.e. diparticle mixtures such as 1:1 (10 nm, 40 nm), 1:1 (10 nm, 70 nm,) and 1:1 (40 nm, 70 nm) were carried out. The results of such diparticle mixtures are shown in Fig. 6b, c and d, respectively.

It can be observed from Fig. 6a, b, c, d that the triparticle mixture and all the diparticle mixtures at 60 ppm result in less than 100 % phenol removal ranging from 70 % for triparticle mixture (Fig. 6a) to 58 % for diparticle (40 nm, 70 nm) (Fig. 6d) for the 10 nm pore membrane. The phenol removal % decreases drastically on higher pore size membranes (M2-M4) for the triparticle and all the diparticle combinations for 60 ppm concentrations. This clearly brings out the effect of particle loss, particle size and the TSA effect. The effect of particle size can be observed from Fig. 6a in that the phenol removal of the triparticle mixture for the  10 nm pore membrane is 70 % however the same is about 80 % for pure 10 nm particles for 60 ppm (Fig. 4a). This indicates mixing the 40 nm & 70 nm particles results  in a net decrease in TSA and so a decrease in phenol removal. The particle size and the mixture effects can be perceived from Figs. 6b to c, in that for the diparticle mixture of 10 nm and 40 nm the phenol removal is again 80 % (Fig. 6b). This result can be explained that without 40 nm particles, there would be some loss of 10 nm particles and in this case that would be minimal as there would be 40 nm particles which would prevent the loss of particles. Hence, the decrease in TSA of pure 60 ppm 10 nm particle is countered by the 40 nm particles to prevent their loss and, additionally, the TSA contribution from the 40 nm particles together might contribut for the equivalent phenol removal % to that of pure 10 nm at 60 ppm silver nanoparticles. The particle loss factor and particle size can also be perceived at high concentrations of 180 ppm from Fig. 6b and c. It can be observed from these Figures that for the diparticle mixture of 10 nm and 40 nm the phenol removal is only about 90 % whereas in the other diparticle mixture of 10 nm and 70 nm it is 100 % for the  80 nm pore membrane. In the former case there would be notable loss of particles, though there might be some agglomeration effect, resulting in less than 100 % phenol removal. However, in the latter case, though there might be loss of 10 nm particles, the contribution of TSA by 70 nm particles coupled with the TSA of 10 nm particles (which are retained by the 70 nm particles and by agglomeration effects) is sufficiently high to attain 100 % phenol retention. Likewise, the combined particle based adsorption effect would be techno-economically and ecologically better for efficient removal of the toxic contents rather than using the individual small and large particles for a given pore size membrane. For example, it can be seen in Figs. 4 and 5 that 100 % phenol removal was not observed even at 180 ppm concentration for 70 nm particles. However, in the case of diparticle mixtures of 10 nm and 70 nm and 40 nm and 70 nm for the same 180 ppm concentration, 100 % phenol removal has been obtained for all pore size membranes including the 80 nm pore size. Though the phenol removal % could be increased with individual particles just by increasing the concentration above 180 ppm, it would not be economical. Similarly, low particle size would be preferred because, generally, it is difficult to synthesize smaller particles and for smaller the particles there are chances that these might interfere with the ecological system. Further, although small particles lead to higher surface areas, it also creates operational issues as small particles would not be stable in a treatment system. They could  be easily swept away by the feed, thus posing difficulties with the maintenance of the system. Hence, for a given set of membranes, a combined particle mixture would yield a better toxic content removal due to the control of the loss of small particles by larger particles by a blocking effect and also by a particle agglomeration effect. This allows for  exploiting the large total surface area (TSA) of small particles.

### COD removal & toxicity study

**Fig. 7:**
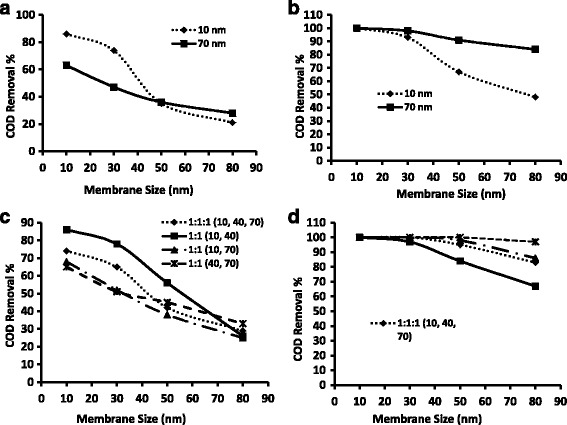
COD removal % through membranes having various average pore sizes for individual particles **a**) 60 ppm, **b**) 180 ppm and for particle mixtures **c**) 60 ppm and **d**) 120 ppm

As a further examination to the phenol removal % study, the filtrates were subjected to chemical oxygen demand (COD) studies as described in the experimental section. For this study, only four selected combinations were chosen from inference of the detailed phenol removal study done in the previous sections. The effect on COD removal by  low and high particle concentrations i.e. 60 ppm and 180 ppm were studied for both individual particle (Fig. 7a & b) and for particle mixtures (Fig. 7c & d). It can be observed from these Figures that the COD removal % almost reflects the phenol removal %. It can be observed from Fig. 7a and b that the 10 nm particle is effective even at 60 ppm particle concentration, but only for the 10 nm & 30 nm membrane pore sizes. Beyond the 30 nm membrane pore size, the COD removal % decreases notable due to particle loss through the larger size pores. With high particle size, i.e. 70 nm, drastic change is not noticed with membrane pore size, there is only a steady decrease. However, with the 180 ppm concentration, such effects are unnoticed due to the high concentration, which results in 100 % COD removal with only a slight decrease for the  50 nm and 80 nm membrane pore size due to phenol and particle loss effects. For the same study particle mixtures, it is interesting to note that the mixture yields a better effect than the results for individual particle sizes. It can be observed from Fig. 6c that the best result is achieved for a 10 nm and 40 nm diparticle mixture. The particle loss effect becomes dominant above the 30 nm membrane pore size. However, it can be observed from the same Figure that the 40 nm and 70 nm diparticle mixture performs better than the other particle mixtures for the 80 nm membrane pore size due to low particle loss and agglomeration effects. With high concentration, i.e. 180 ppm, almost all the diparticle mixtures give close to 100 % COD removal for most membrane pore sizes whereas the triparticle mixture has shows good results only up to the 30 nm membrane pore size and then shows a notable decrease due to the particle loss effects.

To further elucidate the mineralization potential of NANF, toxicity studies with resazurin were conducted for the four combinations for COD removal: the effect of low and high concentrations i.e. 60 ppm and 180 ppm were studied for both the individual particle study (Fig. 7a & b) and also for the particle mixture study (Fig. 7c & d). The results of this study are similar to those observed for the COD reductions and hence the results are not separately presented.

Thus it can be inferred that the small particles perform well even at low concentration for small membrane pore size while the large particles perform well only at high particle concentration for all membrane pore sizes. The diparticle mixture is most  effective in obtaining  good filtration results at low particle concentrations for all the membrane pore sizes.

## Conclusion

This study explores the possibilities of improving toxic chemicals/particles removal through novel nanoparticles assisted nanofiltration (NANF). For this purpose, silver nanoparticles were prepared with three distinct average particle sizes of 10 nm, 40 nm and 70 nm and they were characterized in size and morphology by transmission electron microscopy (TEM). A model phenol compound was prepared and was used for filtration studies through nanoporous membranes with four distinct average pore sizes of 10 nm, 30 nm, 50 nm and 80 nm. This innovative approach yielded as high as 675 % improvement with respect to membrane filtration without nanoparticles. Further the effects of particle size and particle concentrations on filtration were studied. Triparticle mixtures in equal proportion (1:1:1) showed 100 % phenol removal at 180 ppm up to the 50 nm pore size membranes and was reduced to 85 % phenol removal for the 80 nm pore membrane. At 120 ppm, the triparticle mixture showed 100 % phenol removal up to the 30 nm pore size membrane and then decreased notably. The 10 nm and 40 nm diparticle mixture showed > 95 % phenol removal for both 120 ppm and 180 ppm up to the 50 nm pore size membrane and then decreased, whereas 40 nm with 120 ppm particles showed only 80 % phenol removal for the  50 pore membrane. Both the 10 nm & 70 nm and 40 nm & 70 nm diparticle mixtures at 180 ppm showed 100 % phenol removal on all the membranes. The triparticle and diparticle studies reveal  that the particle mixtures result in more phenol removal % than that of the individual particles particularly for small particles for large pore size membranes and larger particles at low particle concentrations. Similar trends were observed both with the COD and toxicity reduction which shows the effectiveness of using particle mixtures. Overall, from this study it can be inferred that NANF has very good potential for treating waste water and the removal of the toxic contents.

## Abbreviations

NANF: nanoparticle assisted nano filtration
M1: membrane with 10 nm pore size
M2: membrane with 30 nm pore size
M3: membrane with 50 nm pore size
M4: membrane with 80 nm pore size
